# ER stress activates lytic gene expression in KSHV-associated tumor cell lines

**DOI:** 10.1186/1750-9378-7-S1-P33

**Published:** 2012-04-19

**Authors:** Nene Kalu, Courtney Shirley, Richard Ambinder

**Affiliations:** 1 Department of Pharmacology and Molecular Sciences, Johns Hopkins School of Medicine, Baltimore, MD, USA; 2 Department of Oncology, Johns Hopkins School of Medicine, Baltimore, MD, USA

## Background

**Figure 1:**
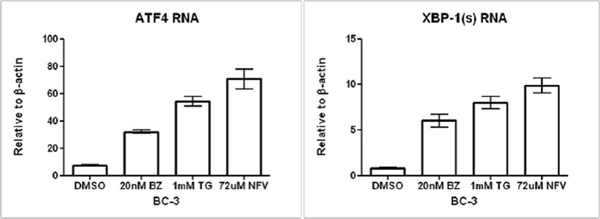
Treatment of BC-3 cells leads to induction of ER stress markers. KSHV PEL cells (BC-3) were treated with DMSO, 20nM bortezomib (20nm BZ), 1mM thapsigargin (1mM TG) or 72uM nelfinavir (72uM NFV). RNA was isolated and RT-PCR was performed using primers for ATF-4 and XBP-1(s). Primers for β-actin were used as an internal control. Error bars indicate SEM.

**Figure 2:**
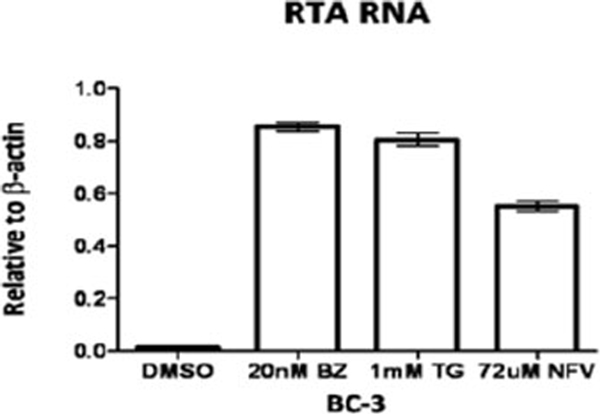
ER stress inducers activate KSHV lytic gene expression. KSHV BC-3 cells were treated with DMSO, 20nM bortezomib (20nm BZ), 1mM thapsigargin (1mM TG) or 72uM nelfinavir. RNA was isolated and RT-PCR was performed using primers for RTA. Primers for β-actin were used as an internal control. Error bars indicate SEM.

Activating the herpesvirus lytic replication cycle presents an opportunity for targeted therapy. We explored the effects of endoplasmic reticulum (ER) stress inducers on Kaposi’s sarcoma herpesvirus (KSHV) lytic activation in primary effusion lymphoma (PEL) cell lines. We included nelfinavir (an HIV-1 protease inhibitor) in our investigations because it has been reported to induce ER stress in various tumor cell lines [1]. Treatment with bortezomib, thapsigargin or nelfinavir resulted in increased expression of ER stress markers such as activating transcription factor 4 (ATF-4) and the spliced form of X-box binding protein 1 (XBP-1(s) (see figure 1). Treatment was also associated with an increase in RNA expression of the KSHV immediate early “replication and transcriptional activator” (RTA) (see figure 2). To determine whether ER stress mediated KSHV lytic reactivation associated with these agents, we prepared doxycycline-activated short hairpin RNA knockdowns of ER stress genes (Grp78 and XBP-1(s)). Treatment of these knockdowns with doxycycline for 72 hours resulted in inhibition of ER stress and inhibition of viral lytic gene expression.

## Conclusion

These results demonstrate that in KSHV-infected cell lines, induction of ER stress is associated with activation of KSHV lytic genes and raises the possibility that nelfinavir might be incorporated into future treatment strategies for KSHV-associated malignancies.
